# Supplementary material for: Integrative Clinical and DNA Methylation Analyses in a Population-Based Cohort Identifies CDH17 and LRP2 as Risk Recurrence Factors in Stage II Colon Cancer
Source: Cancers (Basel). 2022 Dec 27;15(1):158. doi: 10.3390/cancers15010158 (PMC9817957; doi:10.3390/cancers15010158)
Supplement: Supplementary file 1 [file cancers-15-00158-s001.zip › cancers-2128609-supplementary.pdf]

Supplementary Materials and Methods

Integrative Clinical and DNA Methylation Analyses in a Population-Based Cohort Identifies *CDH17* and *LRP2* as Risk Recurrence Factors in Stage II Colon Cancer

Benjamin Tournier [1,2,3], Romain Aucagne [1,2,4,5], Caroline Truntzer [1,2,4,6], Cyril Fournier [1,2,4,5], François Ghiringhelli [1,2,4,5,6], Caroline Chapusot [3], Laurent Martin [1,2,3], Anne Marie Bouvier [1,2], Sylvain Manfredi [1,2,7], Valérie Jooste [1,2,7], Mary B. Callanan [1,2,4,5,*] and Côme Lepage [1,2,7,*]

1 Faculty of health sciences, University of Burgundy, 21000 Dijon, France
2 Institut National de la Santé et de la Recherche Médicale (INSERM) UMR1231, 21000 Dijon, France
3 Department of Pathology, Dijon University Hospital, 21000 Dijon, France
4 Unit for Innovation in Genetics and Epigenetics in Oncology (IGEO) and CRIGEN (Crispr functional genomics), Dijon University Hospital, 21000 Dijon, France
5 Genetics and Immunology Medical Institute, (GIMI), 21000 Dijon, France
6 Centre Georges-François Leclerc (CGFL), 21000 Dijon, France
7 Department of Hepato-Gastroenterology and Digestive oncology, Dijon University Hospital, 21000 Dijon, France
* Correspondence: mary.callanan@u-bourgogne.fr (M.B.C.); come.lepage@u-bourgogne.fr (C.L.)

Supplementary Methods

CIMP analysis.

The CIMP phenotype was determined using a consensus marker panel (Supp. table 1) by methylation specific PCR (193 cases) or by MS-HRM (Methyl Sensitive PCR – High-Resolution Melting curve analysis) for the remaining 190 cases. For 68 samples, results were excluded due to poor quality. MS-HRM PCR were prepared using LightCycler 480 High Resolution Melting Master Mix (Roche) or LightCycler 480 SYBR Green I Master Mix (Roche). Real-time PCR was performed on a LightCycler 480 instrument. Samples were analysed in triplicate and melting curves were compared to methylation standards. Fully methylated genomic DNA (CpGenome Universal Methylated DNA, Merck Millipore) and unmethylated genomic DNA (EpiTect unmethylated Control DNA, Qiagen) were mixed to obtain 50%, 10% and 0% methylated DNA standards. These standards were used to determine the methylation status of CIMP markers. No-CIMP status was defined as no methylated markers, CIMP-Low status as one to three methylated markers, and CIMP-High status as four or five methylated markers.

Chromosomal instability was evaluated by QMPSF.

Chromosomal instability was evaluated using the QMPSF technique (Quantitative Multiplex PCR of Short fluorescent Fragment). This technique was developed by Killian et al. (Gastroenterology, 2007) and investigates the copy number of 8 markers in the genomic DNA, including *TP53* and *EGFR* genes. This technique is patented. We were kindly authorised by the team INSERM "Medical and functional genetics of cancer and neuropsychiatric diseases" unit (Rouen, France) to use this technique. To have access to protocol details, please contact the patent authors. PCR fragments were separated and visualized using an ABI 3130XL sequencer (Applied Biosystems®, Life Technologies). After electrophoresis, the signal heights of each marker fragment were exported in text file format and were gathered in an Excel analysis file (Microsoft® Office Excel®). For each marker, a ratio was calculated by comparing signal height of marker and controls from tumour DNA samples and from corresponding normal mucosa DNA samples. If this ratio was between 0.7 and 1.3, the tumour DNA sample was deemed to have 2 copies of the marker.

If this ratio was less than 0.7, the tumour DNA sample was deemed to have less than 2 copies of the marker. If the ratio was above 1.3, the tumour DNA sample was deemed to have more than 2 copies of the marker.

DNA methylation analyses.

DNA methylation analysis was performed on tumour genomic DNA (2 µg) from 383 patients stage II CC patients by DNA bisulfite conversion and hybridisation onto analysis on GoldenGate methylation array (Illumina) using the Methylation Cancer Panel I, according to manufacturer's instructions. Briefly, the methylation Cancer Panel I investigates a set of 1,505 CpG sites, covering 807 cancer-related genes; tumour suppressor genes, oncogenes, genes involved in DNA repair, cell cycle control, differentiation, apoptosis, X-linked, or imprinted genes. Methylation levels were calculated by dividing signal intensities of methylated alleles by the sum of methylated and unmethylated alleles ((Cy5/Cy5+Cy3 ratio) x 100). These raw values, named beta values, range from 0 for totally unmethylated samples to 1 for totally methylated samples. Pre-processing of array data was performed adjusting to background noise with removal of signals localising to repeats and to the X chromosome and filtering of poor quality data. Final analysis was on 1,026 CpG sites corresponding to 664 genes for 383 patients with colon cancer. For clustering analysis, an unsupervised analysis was performed with the FactoMineR library from R (Lê S et al., Journal of Statistical Software, 2008). This method was used to characterise methylation patterns while introducing no a priori information. It combines a Principal Component Analysis (PCA) with hierarchical clustering (Euclidean metric with Ward method). The optimal number of clusters was chosen to optimise a criterion based on the gain of the within clusters inertia (Husson F et al., Technical Report of the Applied Mathematics Department, 2010). Clusters were defined by the CpG sites which allowed the mean of the cluster to be significantly different from the overall mean which led to a list of 40 CpG sites that identified 4 clusters.

Reanalysis of DNA methylation and RNAseq data from stage II CC from TCGA; validation cohort.

For this study, 295 cases of stage II CC (TCGA) with available Infinium Human Methylation 450K BeadChip (Illumina) were retrieved from the TCGA portal (335 samples, including 40 in duplicate). Of these, 134 samples (116 tumour and 18 adjacent normal tissue samples) from stage II CC cases were selected to validate our methylation classifier. Based on the list of the 40 CpG sites identified by the clustering analysis (see above), a set of 62 CpG sites covering identical or nearest neighbouring site(s) was selected for DNA methylation reanalysis in the TCGA validation cohort (Supp. Table 4). Beta values of this 62 CpG site set were also converted to M-values, as for the original 40 CpG set, using a log2 transformation. The DNA methylation data was then subjected to unsupervised clustering analysis, as above. For 108 of the TCGA stage II CC cases with DNA methylation data, RNA-seq data were also available. RNASeq V2 RSEM normalised expression values were downloaded from Firehose TCGA portal (https://gdac.broadinstitute.org/).

Immunohistochemistry and image analysis.

Sixty formal-fixed and paraffin-embedded tumour (FFPE) tissue blocks were available for the immunohistochemistry analysis. Sections of 4 m thickness were made from the FFPE tissue blocks and the tissue slides were placed on silanised glass slides (SuperFrost®Plus, ThermoScientific). Slides were dried at 56°C for 2 hours. Automated immunohistochemistry (BenchMark ULTRA, Ventana, Roche) was performed using internally validated procedures for detection of the T cell population by using an anti-CD3 antibody (SP7 clone, ThermoScientific). Stained slides were digitalised using a NanoZoomer-HT2.0 slide scanner (Hamamatsu). Only 45 tumour cases displayed sufficient staining quality for the image analysis. Images were analysed using QuPath software (version 0.3.2), as follows: 1. using script editor, 8 squares of 0.46 mm2 each were created in whole slide images (WSI), corresponding to an analysis area of 3.7 mm2. For all WSI, analysis squares (named “annotation”) were manually moved to the tumour core; 2. For the CD3 stain, an automatic normalization of stain vectors was applied. 3. A threshold on optical density sum was used to detect nuclei and a threshold on DAB staining measurement was also

set to determined cell positivity. 4. Using QuPath detection scripting, positive nucleus detection was performed for all analysis squares of each slide. Positive nucleus number per mm^2 was calculated for analysis squares and finally the mean of positive nucleus number per mm^2 for each WSI was calculated.

Supplementary Tables

Supplementary Table S1:

Gene/Marker	Primer	5'→3' sequence
MLH1	Forward	GTCGTAAGGGGAGAGGAG
	Reverse	TCCCTAAAACGACTACTACCC
CDKN2A	Forward	CGTTAGTATCGGAGGAAGAAAGAG
	Reverse	AACGCCCGCACCTCCTCTA
MINT1	Forward	TTTGTTTGGCGTTTAAGAG
	Reverse	CTCTCCCCTCTAAACTTCA
MINT2	Forward	CGTCGTTTATTTTGAAAGTTTGAA
	Reverse	ACACCAAAAAATCACTCCCCTAA
MINT31	Forward	TTTCGTAGATGTTGGGGAAGTGT
	Reverse	CGACGCCACTCCAAAAAACTATA

Supplementary Table S1 | Primer sequences for analysis of CIMP markers by MS-HRM.

Supplementary Table S2:

Marker	Chromosomal band	Repeat type	Normal repeat	Gene involved	5'→3' sequence	
**Reference panel**						
BAT25	Chr.4q12	mono-nucleotide	(T)*25	*KIT*	Forward	FAM-TCGCCTCCAAGAATGTAAGT
					Reverse	GTTTCTTCTGCATTTTAACTATGGCTC
BAT26	Chr.2p21	mono-nucleotide	(A)*27	*MSH2*	Forward	NED-TGACTACTTTTGACTTCAGCC
					Reverse	GTTTCTAACCATTCAACATTTTTAACCC
D2S123	Chr.2p16.3	di-nucleotide	(AC)*21	none	Forward	FAM-AAACAGGATGCCTGCCTTTA
					Reverse	GGACTTTCCACCTATGGGAC
D5S346	Chr.5q22.2	di-nucleotide	(TG)*20	*REEP5*	Forward	PET-ACTCACTCTAGTGATAAATCG
					Reverse	AGCAGATAAGACAGTATTACTAGTT
D17S250	Chr.17q12	di-nucleotide	(GT)*20	none	Forward	PET-GGAAGAATCAAATAGACAAT
					Reverse	GCTGGCCATATATATATTTAAACC
**Supplementary markers**						
NR24	Chr.2q11.1	mono-nucleotide	(T)*23	*ZNF2*	Forward	PET-CCATTGCTGAATTTTACCTC
					Reverse	ATTGTGCCATTGCATTCCAA
TGFBR2	Chr.3p24.1	mono-nucleotide	(A)*10	*TGFBR2*	Forward	FAM-CTTTATTCTGGAAGATGCTGC
					Reverse	GTTTCTGAAGAAAGTCTCACCAGGC
MSH3	Chr.5q14.1	mono-nucleotide	(A)*8	*MSH3*	Forward	NED-AGATGTGAATCCCCTAATCAAGC
					Reverse	GTTTCTACTCCCACAATGCCAATAAAAAT
IGF2R	Chr.6q25.3	mono-nucleotide	(G)*8	*IGF2R*	Forward	PET-AGGTCTCCTGACTCAGAAGC
					Reverse	GTTTCTGCGCTGATAAACCTTATGGC
NR22	Chr.11q24.2	mono-nucleotide	(T)*21	*STT3A*	Forward	VIC-GAGGCTTGTCAAGGACATAA
					Reverse	AATTCTGATGCCATCCAGTT
NR21	Chr.14q11.2	mono-nucleotide	(A)*21	*SLC7A8*	Forward	FAM-TAAATGTATGTCTCCCCTGG
					Reverse	ATTCCTACTCCGCATTCACA
BAX	Chr.19q13.33	mono-nucleotide	(G)*8	*BAX*	Forward	VIC-ATCCAGGATCGAGCAGGGCG
					Reverse	GTTTCTACTCGCTCAGCTTCTTGGTG
D18S58	Chr.18q22.3	di-nucleotide	(GT)*18	none	Forward	FAM-GCTCCCGGCTGGTTTT
					Reverse	GCAGGAAATCGCAGGAACTT

Supplementary Table S2 | Microsatellite markers and corresponding primer sequences.

Supplementary Table S3:

Gene	Exon	Codon	Primer type	5'→3' sequence
KRAS	2	12 and 13	PCR Forward	GGCCTGCTGAAAATGACTGA
			PCR Reverse	Biot-AGCTGTATCGTCAAGGCACTCT
			Pyrosequencing primer	CTTGTGGTAGTTGGAGC
BRAF	15	600	PCR Forward	GGCCAAAAATTTAATCAGTGGAA
			PCR Reverse	Biot-TTCATGAAGACCTCACAGTAAAAA
			Pyrosequencing primer	CCACTCCATCGAGATT
PIK3CA	9	545	PCR Forward	Biot-CTAGCTAGAGACAATGAATTAAGGGAAA
			PCR Reverse	CATTTTAGCACTTACCTGTGACTCCA
			Pyrosequencing primer	CCTGTGACTCCATAGAAAA
PIK3CA	20	1047	PCR Forward	TGAGCAAGAGGCTTTGGAGT
			PCR Reverse	Biot-AAGATCCAATCCATTTTTGTTGTC
			Pyrosequencing primer	CATGAAACAAATGAATGAT

Supplementary Table S3 | Primer sequences for the PCR amplification and subsequent pyrosequencing of *KRAS*, *BRAF* and *PIK3CA*.

Supplementary Table S4:

CpG sites defining Methylation Clusters (Illumina GoldenGate panel)								Equivalent CpG sites (Illumina Infinum panel)							
								Equivalent CpG site n°1				Equivalent CpG site n°2			
Probe ID	cg number	Gene symbol	Gene ID	Chromosome	Genomic position - GRCh37 (hg19)	Dist to TSS	CpG island (Y=YES, N=No)	Ilumina ID	Genomic position - GRCh37 (hg19)	Strand	Distance to site of interest	Ilumina ID	Genomic position - GRCh37 (hg19)	Strand	Distance to site of interest
LRP2_E20_F	cg10022744	LRP2	4036	2	170218993	20	Y	cg13436799	170218997	F	4	cg05660179	170218690	F	-303
CDH17_E31_F	cg14353573	CDH17	1015	8	95220779	31	N	cg17768665	95221197	F	418	cg12038710	95220583	R	-196
LCN2_P86_R	cg24638195	LCN2	3934	9	130911632	-86	N	cg14615559	130911577	F	-55	/	/	/	/
PTHR1_P258_F	cg13804333	PTHR1	5745	3	46918978	-258	N	cg06042608	46918356	R	-622	/	/	/	/
MUSK_P308_F	cg22051739	MUSK	4593	9	113430831	-308	N	cg03689799	113430978	F	147	/	/	/	/
SOD3_P225_F	cg10307548	SOD3	6649	4	24795830	-225	N	cg10307548	24795830	F	0	/	/	/	/
TDGF1_E53_R	cg24195016	TDGF1	6997	3	46619266	53	Y	cg10242476	46619364	R	98	cg16875182	46619291	F	25
ACTG2_P346_F	cg15966469	ACTG2	72	2	74119747	-346	N	cg24691835	74119781	F	34	/	/	/	/
PYCARD_P150_F	cg15468095	PYCARD	29108	16	31214401	-150	Y	cg09115984	31214426	R	25	cg12100791	31214417	R	16
SLC22A2_P109_F	cg21755969	SLC22A2	6582	6	160680068	-109	Y	cg06236276	160680078	R	10	cg21755969	160680068	R	0
RARRES1_P57_R	cg12199224	RARRES1	5918	3	158450332	-57	Y	cg03269060	158450208	R	-124	cg14226182	158450251	R	-81
MAS1_P657_R	cg13390345	MAS1	4142	6	160327317	-657	N	cg02582754	160327345	R	28	/	/	/	/
TBX1_P520_F	cg15095600	TBX1	6899	22	19743706	-520	N	cg02948624	19743759	F	53	cg26520942	19743892	R	186
LCK_E28_F	cg05350315	LCK	3932	1	32716961	28	Y	cg05350315	32716961	R	0	/	/	/	/
IL12B_P1453_F	cg11461541	IL12B	3593	5	158758934	-1453	Y	cg07622001	158758903	R	-31	/	/	/	/
CDKN1C_P6_R	cg08331513	CDKN1C	1028	11	2906981	-6	Y	cg08331513	2906981	F	0	cg05989775	2906934	F	-47
VEGFB_P658_F	cg03028312	VEGFB	7423	11	64001608	-658	Y	cg03057079	64001712	R	104	/	/	/	/
p16_seq_47_S85_F	cg17449661	CDKN2A	1029	9	21974812	.	Y	cg13601799	21974704	F	-108	/	/	/	/
TDG_E129_F	cg09857351	TDG	6996	12	104359746	129	Y	cg03923277	104359732	R	-14	/	/	/	/
GNMT_P197_F	cg04013093	GNMT	27232	6	42928303	-197	Y	cg04013093	42928303	R	0	cg11409096	42928337	R	34
ACVR1C_P115_R	cg19905951	ACVR1C	130399	2	158485514	-115	Y	cg17868920	158485541	R	27	cg18004523	158485526	R	12

BCL3_E71_F	cg21016032	BCL3	602	19	45252102	71	Y	cg16632661	45251986	F	-116	/	/	/	/
FASTK_P598_R	cg22085103	FASTK	10922	7	150778549	-598	Y	cg11823624	150778724	F	175	cg18068487	150778340	R	-209
HDAC5_E298_F	cg08753986	HDAC5	10014	17	42200716	298	Y	cg17842157	42200988	F	272	/	/	/	/
MST1R_E42_R	cg03714052	MST1R	4486	3	49941028	42	Y	cg03332271	49941151	F	123	cg11839681	49940919	R	-109
CRIP1_P874_R	cg03324382	CRIP1	1396	14	105952675	-874	Y	cg07065217	105953092	F	417	cg26110883	105953088	F	413
NBL1_P24_F	cg04102045	NBL1	4681	1	19969702	-24	N	cg21813747	19969931	R	229	cg21057046	19970204	F	502
APBA2_P305_R	cg17121025	APBA2	321	15	29213548	-305	N	cg08908089	29213250	R	-298	cg20840847	29213626	F	78
SFN_E118_F	cg08545357	SFN	2810	1	27189751	118	Y	cg13466284	27189679	F	-72	cg03421300	27189787	R	36
SERPINB5_P19_R	cg22362057	SERPINB5	5268	18	61144200	-19	Y	cg20837735	61144177	R	-23	cg08411049	61144250	F	50
TMPRSS4_E83_F	cg13233515	TMPRSS4	56649	11	117947849	83	N	cg25116503	117947657	R	-192	/	/	/	/
NTRK3_E131_F	cg00865584	NTRK3	4916	15	88799530	131	Y	cg27034819	88799526	F	-4	cg11525479	88799523	F	-7
EPHA7_P205_R	cg24188617	EPHA7	2045	6	94129477	-205	Y	cg08734918	94129481	F	4	cg03696441	94129431	R	-46
GAS7_E148_F	cg20649212	GAS7	8522	17	10101720	148	Y	cg25116216	10101581	F	-139	/	/	/	/
WT1_E32_F	cg20134916	WT1	7490	11	32457055	32	Y	cg07193766	32457127	R	72	cg13663793	32457124	R	69
FLT3_P302_F	cg23603794	FLT3	2322	13	28675007	-302	Y	cg09400887	28675056	F	49	/	/	/	/
HIC2_P498_F	cg21597330	HIC2	23119	22	21771195	-498	Y	cg13558199	21770821	F	-374	/	/	/	/
PROK2_P390_F	cg01645467	PROK2	60675	3	71834602	-390	Y	cg08555612	71834640	F	38	/	/	/	/
VIM_P343_R	cg07185035	VIM	7431	10	17270955	-343	Y	cg11973177	17271006	F	51	cg26983469	17271051	F	96
PDE1B_P263_R	cg06901488	PDE1B	5153	12	54943141	-263	Y	cg13565157	54943009	F	-132	cg00639886	54943102	F	-39

Supplementary Table S4 | Comparison of GoldenGate and Infinium CpG sites

On the left side of the table are indicated (rows), the CpG sites comprising the MethylColon II classifier and their genome position, from the GoldenGate panel. On the right side of the table are indicated the equivalent CpG sites from the Infinium panel (62 CpG sites), their genome position and their distance (in nucleotides) from the GoldenGate CpG sites. For 22 CpG sites, 2 equivalent CpG sites were selected from the Infinium panel and for 18 CpG sites, 1 CpG site was selected.

Supplementary Table S5:

	**Univariate models**		**Multivariate models**	
CpG sites	Chi2	*P* value	Chi2	*P* value
SLC22A2_P109_F	2.10	0.5520		
RARRES1_P57_R	1.41	0.7023		
MAS1_P657_R	1.62	0.6560		
TBX1_P520_F	0.28	0.9636		
LCK_E28_F	1.80	0.6158		
GNMT_P197_F	3.81	0.2822		
ACVR1C_P115_R	2.35	0.5026		
BCL3_E71_F	2.57	0.4620		
FASTK_P598_R	2.74	0.4340		
HDAC5_E298_F	3.49	0.3220		
APBA2_P305_R	5.03	0.1693		
LCN2_P86_R	11.19	**0.0108**	0.24	0.9717
SFN_E118_F	10.48	**0.0149**	6.01	0.1110
SERPINB5_P19_R	5.02	0.1702		
TMPRSS4_E83_F	7.74	0.0517		
LRP2_E20_F	17.07	**0.0007**	12.97	**0.0047**
HIC2_P498_F	3.76	0.2887		
PROK2_P390_F	2.12	0.5481		
VIM_P343_R	2.28	0.5172		
PDE1B_P263_R	5.88	0.1177		
MUSK_P308_F	7.74	0.0516		
SOD3_P225_F	7.35	0.0616		
TDGF1_E53_R	3.64	0.3026		
ACTG2_P346_F	1.04	0.7911		
PYCARD_P150_F	2.14	0.5445		
IL12B_P1453_F	4.42	0.2197		
CDKN1C_P6_R	4.17	0.2437		
VEGFB_P658_F	0.51	0.9163		
p16_seq_47_S85_F	0.63	0.8896		
TDG_E129_F	3.13	0.3716		
CDH17_E31_F	14.26	**0.0026**	9.80	**0.0203**
MST1R_E42_R	8.00	**0.0461**	6.26	0.0998
PTHR1_P258_F	11.29	**0.0102**	5.87	0.1184
CRIP1_P874_R	3.71	0.2950		
NBL1_P24_F	6.09	0.1075		
NTRK3_E131_F	1.77	0.6225		
EPHA7_P205_R	2.13	0.5462		
GAS7_E148_F	3.42	0.3319		
WT1_E32_F	1.00	0.8017		
FLT3_P302_F	1.64	0.6492		

Supplementary Table S5 | Univariate and multivariate relapse analyses in stage II colon cancer patients. Significant markers are in grey.

Supplementary Table S6:

GENE SET	SIZE	ES	NES	NOM p-val	FDR q-val	FWER p-val	RANK AT MAX
HALLMARK_IL2_STAT5_SIGNALING	194	0.64076215	1.6851475	0.0	0.22216089	0.09	3412
HALLMARK_APICAL_SURFACE	43	0.6695254	1.6632961	0.0	0.13807619	0.106	3161
HALLMARK_APOPTOSIS	158	0.5973283	1.6237148	0.0019267823	0.13683935	0.152	4232
HALLMARK_COMPLEMENT	195	0.6826357	1.614319	0.0	0.11707509	0.164	2824
HALLMARK_KRAS_SIGNALING_UP	192	0.6828222	1.5934346	0.0	0.118327916	0.208	2494
HALLMARK_ALLOGRAFT_REJECTION	195	0.7308803	1.5899248	0.009633912	0.10176944	0.214	3263
HALLMARK_INFLAMMATORY_RESPONSE	197	0.72928864	1.5710777	0.003976143	0.10763645	0.256	3097
HALLMARK_APICAL_JUNCTION	193	0.6166698	1.56364	0.009881423	0.10048622	0.269	3224
HALLMARK_MYOGENESIS	198	0.6628489	1.5592813	0.011952192	0.09277076	0.279	3352
HALLMARK_TNFA_SIGNALING_VIA_NFKB	197	0.68563247	1.5533555	0.03	0.089052774	0.289	3609
HALLMARK_COAGULATION	135	0.6675979	1.5276564	0.020637898	0.09788468	0.342	2538
HALLMARK_INTERFERON_GAMMA_RESPONSE	196	0.7450341	1.5217805	0.01953125	0.09468812	0.354	3342
HALLMARK_IL6_JAK_STAT3_SIGNALING	87	0.70341784	1.4931202	0.015686275	0.11494249	0.421	2753
HALLMARK_HYPOXIA	190	0.5444028	1.4639924	0.025490196	0.13591139	0.482	4078
HALLMARK_UV_RESPONSE_UP	152	0.4441565	1.4288329	0.017578125	0.15465479	0.556	3494
HALLMARK_EPITHELIAL_MESENCHYMAL_TRANSITION	194	0.79196525	1.4201883	0.03529412	0.1553128	0.573	2614
HALLMARK_HEME_METABOLISM	189	0.39115623	1.3719858	0.04771372	0.17777409	0.676	4994
HALLMARK_ESTROGEN_RESPONSE_EARLY	192	0.47889447	1.3389112	0.046464648	0.1973141	0.735	3384

Supplementary Table S6 | Hallmark gene sets enriched in hypermethylated *CDH17* stage II CC.
This table contains the most significant, positively-enriched pathways in the hypermethylated *CDH17* (the 25% highest beta values for the cg17768665 CpG site; Q4), stage II CC tumour subgroup as compared to all other cases (validation cohort, TCGA; see main article for details), by GSEA analysis against "Hallmark" gene sets (H), as indicated. Abbreviations as follows: enrichment scores (ES), normalised enrichment scores (NES), nominal p values (NOM p-val) and false discovery rates (FDR q-val).

Supplementary Table S7:

GENE SET	SIZE	ES	NES	NOM p-val	FDR q-val	FWER p-val	RANK AT MAX
HALLMARK_E2F_TARGETS	187	0.6803183	1.5733525	0.031894933	0.101083554	0.233	3606
HALLMARK_MTORC1_SIGNALING	192	0.55778146	1.6874044	0.01171875	0.11174072	0.065	3745
HALLMARK_DNA_REPAIR	140	0.50685644	1.6002958	0.021400778	0.12621935	0.188	4692
HALLMARK_REACTIVE_OXYGEN_SPECIES_PATHWAY	45	0.5126451	1.5180712	0.02886598	0.13696657	0.345	4890
HALLMARK_G2M_CHECKPOINT	183	0.6023733	1.6102422	0.031657357	0.17152718	0.173	3256

Supplementary Table S7 | Hallmark gene sets enriched in hypermethylated *LRP2* stage II CC.
This table contains the most significant positively-enriched pathways in the hypermethylated *LRP2* (the 25% highest beta values for the cg13436799 CpG site; Q4) stage II CC tumour subgroup, as compared to all other cases (validation cohort, TCGA; see main article for details), by GSEA analysis against "Hallmark" gene sets (H), as indicated. Abbreviations as follows: enrichment scores (ES), normalised enrichment scores (NES), nominal p values (NOM p-val) and false discovery rates (FDR q-val).

Supplementary Table S8:

GENE SET	SIZE	ES	NES	NOM p-val	FDR q-val	FWER p-val	RANK AT MAX
GSE36476_CTRL_VS_TSST_ACT_40H_MEMORY_CD4_TCELL_OLD_DN	185	0.6874494	1.800827	0.0061099795	0.21442017	0.125	3766
GSE13547_CTRL_VS_ANTI_IGM_STIM_BCELL_12H_UP	174	0.68116474	1.7978802	0.012	0.16580273	0.126	3256
GSE28726_NAIVE_CD4_TCELL_VS_NAIVE_VA24NEG_NKTCELL_UP	190	0.6437267	1.7937709	0.001996008	0.14018527	0.133	2895
GSE14415_INDUCED_VS_NATURAL_TREG_DN	169	0.65589756	1.7935144	0.0060728746	0.1173619	0.133	3447
GSE13547_2H_VS_12_H_ANTI_IGM_STIM_ZFX_KO_BCELL_DN	159	0.6135387	1.7910873	0.002020202	0.104120165	0.138	3715
GSE36476_CTRL_VS_TSST_ACT_40H_MEMORY_CD4_TCELL_YOUNG_DN	185	0.6832061	1.7827239	0.008130081	0.10118296	0.151	3409
GSE3982_NKCELL_VS_TH2_DN	180	0.60375464	1.7777342	0.008080808	0.0991646	0.167	2969
GSE13547_CTRL_VS_ANTI_IGM_STIM_ZFX_KO_BCELL_2H_UP	148	0.5850074	1.7775521	0.005952381	0.09020182	0.169	4196
GSE26156_DOUBLE_POSITIVE_VS_CD4_SINGLE_POSITIVE_THYMOCYTE_DN	188	0.6148029	1.7713209	0.0040983604	0.08921193	0.178	4120
GSE24574_BCL6_LOW_TFH_VS_TCONV_CD4_TCELL_DN	186	0.619197	1.7599903	0.003984064	0.09762585	0.208	3789
GSE33162_UNTREATED_VS_4H_LPS_STIM_HDAC3_KO_MACROPHAGE_DN	187	0.56606257	1.753226	0.007968128	0.09867604	0.223	3606
GSE14415_NATURAL_TREG_VS_TCONV_DN	167	0.6285158	1.7457064	0.012072435	0.100482546	0.236	3256
GSE14415_TCONV_VS_FOXP3_KO_INDUCED_TREG_DN	166	0.6544377	1.7428685	0.008247423	0.09581105	0.239	3447
GSE21927_BALBC_VS_C57BL6_MONOCYTE_TUMOR_UP	186	0.60209805	1.7420294	0.0020120724	0.09039247	0.24	4919
GSE24634_TEFF_VS_TCONV_DAY7_IN_CULTURE_UP	188	0.637301	1.741327	0.011976048	0.08614048	0.242	2951
GSE14415_INDUCED_TREG_VS_TCONV_UP	158	0.6699448	1.7377895	0.012024048	0.08549248	0.245	4358
GSE36476_CTRL_VS_TSST_ACT_72H_MEMORY_CD4_TCELL_OLD_DN	180	0.6558346	1.7252452	0.012096774	0.094894454	0.271	3789
GSE13547_2H_VS_12_H_ANTI_IGM_STIM_BCELL_DN	164	0.67992574	1.7228727	0.012145749	0.093299195	0.277	4323
GSE17974_0H_VS_48H_IN_VITRO_ACT_CD4_TCELL_DN	170	0.5182705	1.7109182	0.015533981	0.102752864	0.303	4840
GSE21063_WT_VS_NFATC1_KO_8H_ANTI_IGM_STIM_BCELL_UP	176	0.6623497	1.7101579	0.015873017	0.098765776	0.306	3409
GSE32986_UNSTIM_VS_CURDLAN_HIGHDOSE_STIM_DC_UP	179	0.50711834	1.7075014	0.007920792	0.097684294	0.318	3421
GSE10239_NAIVE_VS_DAY4.5_EFF_CD8_TCELL_DN	177	0.606371	1.7070686	0.014198783	0.094296984	0.32	3053
GSE36826_WT_VS_IL1R_KO_SKIN_STAPH_AUREUS_INF_UP	184	0.5586493	1.7060543	0.0059405942	0.09237098	0.326	3764
KAECH_DAY8_EFF_VS_MEMORY_CD8_TCELL_UP	181	0.59202296	1.704796	0.01004016	0.09037501	0.329	3415
GSE22886_UNSTIM_VS_IL15_STIM_NKCELL_DN	183	0.64969045	1.7040335	0.015968064	0.08820222	0.332	4209
GSE41867_LCMV_ARMSTRONG_VS_CLONE13_DAY6_EFFECTOR_CD8_TCELL_UP	175	0.5184379	1.7032101	0.004016064	0.08644704	0.334	4215
GSE14415_INDUCED_TREG_VS_FAILED_INDUCED_TREG_UP	174	0.61758494	1.6957704	0.01968504	0.09257124	0.356	4340
GSE32986_UNSTIM_VS_GMCSF_AND_CURDLAN_LOWDOSE_STIM_DC_UP	184	0.5508998	1.6896629	0.008368201	0.096609674	0.375	3413
GSE24634_TREG_VS_TCONV_POST_DAY7_IL4_CONVERSION_UP	180	0.60879344	1.6865271	0.016	0.09712783	0.382	3011
GSE17974_0H_VS_72H_IN_VITRO_ACT_CD4_TCELL_DN	174	0.5244293	1.6855115	0.01010101	0.09533792	0.39	3515
GSE3982_CENT_MEMORY_CD4_TCELL_VS_TH1_DN	183	0.58785343	1.6845255	0.0061099795	0.093469016	0.393	3978
GSE11386_NAIVE_VS_MEMORY_BCELL_UP	168	0.5330004	1.6793858	0.015810277	0.097423375	0.408	4392
GSE36476_CTRL_VS_TSST_ACT_72H_MEMORY_CD4_TCELL_YOUNG_DN	181	0.6517192	1.6779432	0.012121212	0.09659983	0.412	2951

GSE21927_SPLEEN_C57BL6_VS_EL4_TUMOR_BALBC_MONOCYTES_DN	181	0.5968493	1.6755679	0.02004008	0.09695643	0.418	4539
GSE17974_CTRL_VS_ACT_IL4_AND_ANTI_IL12_24H_CD4_TCELL_DN	177	0.59303814	1.6678704	0.012048192	0.10481789	0.446	4728
GSE8685_IL2_STARVED_VS_IL2_ACT_IL2_STARVED_CD4_TCELL_DN	183	0.46243888	1.6664934	0.0063157897	0.10393449	0.449	3387
GOLDRATH_EFF_VS_MEMORY_CD8_TCELL_UP	189	0.6273593	1.6662623	0.014285714	0.101652615	0.449	3282
GSE24634_NAIVE_CD4_TCELL_VS_DAY7_IL4_CONV_TREG_DN	184	0.53457624	1.6630958	0.01953125	0.10340963	0.459	2883
GSE21927_GMCSF_IL6_VS_GMCSF_GCSF_TREATED_BONE_MARROW_DN	178	0.54897845	1.6628023	0.022494888	0.10115473	0.46	4032
GSE39110_DAY3_VS_DAY6_POST_IMMUNIZATION_CD8_TCELL_WITH_IL2_TREATMENT_UP	186	0.5322535	1.6583972	0.012145749	0.1049681	0.47	4563
GSE5679_CTRL_VS_PPARG_LIGAND_ROSIGLITAZONE_TREATED_DC_UP	174	0.6326482	1.6583827	0.018518519	0.10252699	0.47	3785
GSE17974_0.5H_VS_72H_UNTREATED_IN_VITRO_CD4_TCELL_DN	170	0.53039104	1.6575618	0.016260162	0.10115933	0.472	3282
GSE22886_NAIVE_CD4_TCELL_VS_48H_ACT_TH1_DN	180	0.57205427	1.6548862	0.025896415	0.102426395	0.48	4289
GSE22886_UNSTIM_VS_IL2_STIM_NKCELL_DN	180	0.61141443	1.6542695	0.02008032	0.101104125	0.485	4099
GSE22611_NOD2_TRANSDUCED_VS_CTRL_HEK293T_STIMULATED_WITH_MDP_2H_DN	170	0.4824594	1.6538843	0.008	0.099503055	0.485	3448
GSE24634_TREG_VS_TCONV_POST_DAY5_IL4_CONVERSION_UP	179	0.59848464	1.6530647	0.023622047	0.09853152	0.492	3889
GSE43863_TH1_VS_TFH_MEMORY_CD4_TCELL_UP	178	0.4861171	1.6526238	0.006134969	0.09723082	0.493	3394
KAECH_DAY8_EFF_VS_DAY15_EFF_CD8_TCELL_UP	179	0.5707971	1.6520528	0.026156941	0.095805414	0.494	3849
GSE5679_CTRL_VS_RARA_AGONIST_AM580_TREATED_DC_UP	179	0.5945265	1.6480898	0.01968504	0.099372536	0.504	4073
GSE7568_IL4_VS_IL4_AND_DEXAMETHASONE_TREATED_MACROPHAGE_UP	151	0.55445	1.6477545	0.029880479	0.09794928	0.505	4342
GSE3982_MEMORY_CD4_TCELL_VS_TH2_DN	184	0.5608242	1.644571	0.02244898	0.09991898	0.512	3544
GSE15750_DAY6_VS_DAY10_TRAF6KO_EFF_CD8_TCELL_UP	179	0.6486463	1.641597	0.038240917	0.10220932	0.526	3919
GSE43863_TH1_VS_LY6C_LOW_CXCR5NEG_EFFECTOR_CD4_TCELL_UP	174	0.5249494	1.6407238	0.016	0.10148248	0.528	3307
GSE40274_CTRL_VS_FOXP3_AND_XBP1_TRANSDUCED_ACTIVATED_CD4_TCELL_UP	151	0.5115702	1.6383713	0.001996008	0.102970175	0.54	2736
GSE3337_4H_VS_16H_IFNG_IN_CD8POS_DC_UP	187	0.5146763	1.6365474	0.01026694	0.10375287	0.548	4621
GSE43863_NAIVE_VS_MEMORY_TFH_CD4_TCELL_D150_LCMV_DN	179	0.49803752	1.6312863	0.0063157897	0.109525464	0.566	3278
GSE2405_0H_VS_9H_A_PHAGOCYTOPHILUM_STIM_NEUTROPHIL_DN	180	0.6238642	1.6302215	0.040816326	0.10894722	0.569	5155
GSE15930_NAIVE_VS_24H_IN_VITRO_STIM_INFAB_CD8_TCELL_DN	186	0.57224154	1.6291362	0.03137255	0.10868	0.571	3837
GSE15930_NAIVE_VS_72H_IN_VITRO_STIM_IFNAB_CD8_TCELL_DN	181	0.54475474	1.6250185	0.01996008	0.112565786	0.579	3814
GSE36476_CTRL_VS_TSST_ACT_16H_MEMORY_CD4_TCELL_OLD_DN	184	0.5021601	1.624188	0.020366598	0.11216099	0.584	3791
GSE15930_NAIVE_VS_48H_IN_VITRO_STIM_IL12_CD8_TCELL_DN	181	0.54951555	1.6231123	0.025742574	0.11208193	0.588	3749
GOLDRATH_NAIVE_VS_EFF_CD8_TCELL_DN	187	0.61288047	1.6152124	0.011904762	0.12249318	0.607	3736
GSE24634_TEFF_VS_TCONV_DAY5_IN_CULTURE_UP	183	0.5897964	1.6149281	0.04771372	0.121222906	0.609	3635
GSE13547_WT_VS_ZFX_KO_BCELL_ANTI_IGM_STIM_2H_UP	166	0.55927366	1.6148562	0.042510122	0.11957978	0.609	3583
GSE35543_IN_VITRO_ITREG_VS_CONVERTED_EX_ITREG_UP	174	0.5420721	1.6105632	0.021868788	0.12462697	0.623	3442
GSE15750_DAY6_VS_DAY10_EFF_CD8_TCELL_UP	177	0.65486056	1.6066763	0.036	0.12923008	0.632	3256
GSE40274_CTRL_VS_FOXP3_TRANSDUCED_ACTIVATED_CD4_TCELL_UP	176	0.5445109	1.6037465	0.025948104	0.13259345	0.646	3583
GSE3982_NKCELL_VS_TH1_DN	183	0.5224028	1.6032847	0.01629328	0.13149132	0.647	2969
GSE7852_LN_VS_THYMUS_TCONV_DN	178	0.46886712	1.6030177	0.00811359	0.13011032	0.648	2758
GSE17974_CTRL_VS_ACT_IL4_AND_ANTI_IL12_72H_CD4_TCELL_DN	168	0.46550786	1.6027471	0.005988024	0.1286923	0.648	4340
GSE1460_INTRATHYMIC_T_PROGENITOR_VS_NAIVE_CD4_TCELL_ADULT_BLOOD_UP	184	0.4918396	1.602656	0.020576132	0.12705429	0.648	3597

GSE22886_UNSTIM_VS_STIM_MEMORY_TCELL_DN	183	0.53604764	1.6024376	0.032323234	0.12567073	0.648	4197
GSE17974_0H_VS_24H_IN_VITRO_ACT_CD4_TCELL_DN	173	0.5457304	1.5980752	0.020618556	0.13095187	0.661	3777
GSE5142_HTERT_TRANSDUCED_VS_CTRL_CD8_TCELL_LATE_PASSAGE_CLONE_UP	173	0.5472761	1.5975462	0.031809144	0.13001788	0.663	4053
GSE32986_UNSTIM_VS_GMCSF_AND_CURDLAN_HIGHDOSE_STIM_DC_UP	176	0.47991577	1.5968384	0.013833992	0.12934926	0.664	2941
GSE28726_ACT_CD4_TCELL_VS_ACT_VA24NEG_NKTCELL_UP	188	0.46167612	1.5957853	0.028282829	0.12923923	0.669	4192
GSE12845_NAIVE_VS_PRE_GC_TONSIL_BCELL_DN	184	0.4783613	1.5943145	0.025793651	0.12989597	0.67	4433
GSE23114_PERITONEAL_CAVITY_B1A_BCELL_VS_SPLEEN_BCELL_DN	184	0.45713195	1.5930934	0.014403292	0.13008091	0.674	4209
GSE15930_NAIVE_VS_24H_IN_VITRO_STIM_IL12_CD8_TCELL_DN	187	0.5467412	1.589114	0.046875	0.13499048	0.687	4602
GSE22886_NEUTROPHIL_VS_MONOCYTE_DN	177	0.467019	1.5861801	0.017509727	0.13858289	0.694	4675
GSE28726_NAIVE_VS_ACTIVATED_VA24NEG_NKTCELL_UP	185	0.46141246	1.5859374	0.015533981	0.13742101	0.695	4610
GSE30962_PRIMARY_VS_SECONDARY_ACUTE_LCMV_INF_CD8_TCELL_UP	186	0.5778244	1.5856042	0.043392505	0.13644436	0.695	3256
GSE3982_MEMORY_CD4_TCELL_VS_TH1_DN	183	0.5421288	1.5840399	0.020576132	0.13776237	0.699	3544
GSE3982_CENT_MEMORY_CD4_TCELL_VS_TH2_DN	184	0.5469208	1.5836117	0.015904572	0.13674466	0.7	4273
GSE9650_EFFECTOR_VS_MEMORY_CD8_TCELL_UP	185	0.51990515	1.580736	0.008064516	0.13981505	0.707	3920
GSE22886_NAIVE_CD4_TCELL_VS_48H_ACT_TH2_DN	181	0.50806594	1.5774777	0.043564357	0.14401983	0.715	4085
GSE15930_NAIVE_VS_24H_IN_VITRO_STIM_CD8_TCELL_DN	187	0.55487424	1.5773008	0.043392505	0.14270462	0.715	3412
GSE27786_NEUTROPHIL_VS_MONO_MAC_DN	178	0.42127368	1.577125	0.001980198	0.1413747	0.716	4560
GSE24634_NAIVE_CD4_TCELL_VS_DAY3_IL4_CONV_TREG_DN	182	0.48938394	1.5767401	0.032786883	0.14030671	0.717	4291
GSE39110_DAY3_VS_DAY6_POST_IMMUNIZATION_CD8_TCELL_DN	177	0.55090237	1.5746784	0.035433073	0.14267078	0.726	3256
GSE19941_LPS_VS_LPS_AND_IL10_STIM_IL10_KO_MACROPHAGE_UP	162	0.47939673	1.5722328	0.031067962	0.14562751	0.731	3911
GSE45739_NRAS_KO_VS_WT_ACD3_ACD28_STIM_CD4_TCELL_UP	173	0.47170585	1.5722151	0.017094018	0.14410746	0.731	4554
GSE28408_LY6G_POS_VS_NEG_DC_DN	176	0.51077694	1.5709898	0.010224949	0.1445605	0.735	4251
GSE21546_UNSTIM_VS_ANTI_CD3_STIM_DP_THYMOCYTES_DN	171	0.49964496	1.5662261	0.037698414	0.15180483	0.745	4701
GSE28726_ACT_CD4_TCELL_VS_ACT_NKTCELL_UP	187	0.4532011	1.565912	0.014373717	0.15087593	0.746	4770
GSE41867_DAY8_VS_DAY15_LCMV_CLONE13_EFFECTOR_CD8_TCELL_DN	177	0.4836522	1.5658665	0.03256705	0.14945515	0.746	3849
GSE16451_CTRL_VS_WEST_EQUINE_ENC_VIRUS_IMMATURE_NEURON_CELL_LINE_DN	178	0.47640952	1.5652057	0.0140280565	0.14925085	0.748	4099
GSE12001_MIR223_KO_VS_WT_NEUTROPHIL_UP	160	0.43446594	1.5631201	0.018255578	0.15173459	0.753	4684
GSE3720_UNSTIM_VS_PMA_STIM_VD2_GAMMADELTA_TCELL_UP	157	0.48015603	1.5626478	0.01980198	0.15127976	0.754	3904
GSE3982_MAST_CELL_VS_TH2_DN	180	0.46376425	1.5619968	0.016161617	0.15092228	0.755	3870
GSE28237_FOLLICULAR_VS_EARLY_GC_BCELL_DN	184	0.4449742	1.5614251	0.02366864	0.15049326	0.757	4771
GSE24574_NAIVE_VS_TCONV_CD4_TCELL_DN	189	0.4612794	1.5614243	0.040650405	0.14904621	0.757	4209
GSE40274_CTRL_VS_EOS_TRANSDUCED_ACTIVATED_CD4_TCELL_UP	162	0.5404181	1.5600176	0.036363635	0.1501448	0.762	4341
GSE24634_TEFF_VS_TCONV_DAY3_IN_CULTURE_UP	183	0.5293223	1.5579369	0.030737706	0.15236475	0.767	4209
GSE18203_CTRL_VS_INTRATUMORAL_CPG_INJ_MC38_TUMOR_DN	182	0.5024452	1.5575954	0.008385744	0.1514238	0.768	3334
GSE15930_NAIVE_VS_72H_IN_VITRO_STIM_CD8_TCELL_DN	182	0.4913135	1.5533714	0.02970297	0.15738367	0.775	3870
GSE3982_DC_VS_TH1_DN	178	0.49835357	1.5495101	0.014613778	0.16325875	0.785	2484
GSE41867_NAIVE_VS_DAY30_LCMV_ARMSTRONG_MEMORY_CD8_TCELL_DN	175	0.45087188	1.5489751	0.022177419	0.16290396	0.786	5227
GSE22432_CONVENTIONAL_CDC_VS_PLASMACYTOID_PDC_UP	173	0.4569714	1.5487407	0.03285421	0.16186133	0.787	3773

GSE10239_NAIVE_VS_KLRG1INT_EFF_CD8_TCELL_DN	181	0.48491758	1.548682	0.021782178	0.16052958	0.787	3715
GSE13522_CTRL_VS_T_CRUZI_Y_STRAIN_INF_SKIN_BALBC_MOUSE_UP	121	0.45769396	1.5475653	0.013752456	0.16123796	0.79	2902
GSE14415_TCONV_VS_FOXP3_KO_INDUCED_TREG_UP	154	0.4263101	1.5465419	0.031620555	0.1618147	0.792	4350
GSE28726_NAIVE_VS_ACTIVATED_CD4_TCELL_DN	189	0.5375573	1.5465103	0.024590164	0.16045074	0.792	3409
GSE40274_CTRL_VS_FOXP3_AND_HELIOS_TRANSDUCED_ACTIVATED_CD4_TCELL_DN	177	0.43301898	1.5463785	0.016032064	0.15931308	0.793	4189
GSE15624_CTRL_VS_3H_HALOFUGINONE_TREATED_CD4_TCELL_DN	143	0.4300998	1.5431446	0.021868788	0.16289566	0.798	5120
GSE8921_UNSTIM_VS_TLR1_2_STIM_MONOCYTE_3H_UP	178	0.4185321	1.5423342	0.007952286	0.16296521	0.798	3845
GSE33292_WT_VS_TCF1_KO_DN3_THYMOCYTE_DN	191	0.5600335	1.5401428	0.03877551	0.16549894	0.802	3627
GSE10239_NAIVE_VS_KLRG1HIGH_EFF_CD8_TCELL_DN	183	0.5066337	1.5399797	0.025390625	0.16448927	0.802	3697
GSE23505_IL6_IL1_IL23_VS_IL6_IL1_TGFB_TREATED_CD4_TCELL_DN	174	0.44528434	1.5377961	0.011764706	0.16730459	0.806	4532
GSE32164_ALTERNATIVELY_ACT_M2_VS_CMYC_INHIBITED_MACROPHAGE_DN	189	0.47136924	1.5370334	0.027290449	0.1674377	0.809	2934
GSE20715_0H_VS_48H_OZONE_LUNG_DN	190	0.49206415	1.5353345	0.006355932	0.16923073	0.813	3113
GSE23568_ID3_KO_VS_WT_CD8_TCELL_UP	181	0.5183065	1.5340201	0.028688524	0.17028922	0.817	3527
GSE39110_UNTREATED_VS_IL2_TREATED_CD8_TCELL_DAY3_POST_IMMUNIZATION_DN	180	0.4435067	1.5302114	0.015625	0.17522451	0.829	3326
GSE20500_CTRL_VS_RARA_ANTAGONIST_TREATED_CD4_TCELL_DN	173	0.40552336	1.5239491	0.018108651	0.18657741	0.84	2989
GSE20727_CTRL_VS_ROS_INH_AND_DNFB_ALLERGEN_TREATED_DC_DN	172	0.45400286	1.5235958	0.024048096	0.18588805	0.84	4174
GSE14699_DELETIONAL_TOLERANCE_VS_ACTIVATED_CD8_TCELL_DN	163	0.5268191	1.5227749	0.039337475	0.18633792	0.843	4434
GSE20727_DNFB_ALLERGEN_VS_ROS_INH_AND_DNFB_ALLERGEN_TREATED_DC_DN	180	0.46387228	1.5215658	0.009784736	0.18624553	0.846	3422
GSE33162_UNTREATED_VS_4H_LPS_STIM_HDAC3_KO_MACROPHAGE_UP	184	0.48776582	1.5200106	0.021484375	0.18662092	0.852	3746
GSE17974_CTRL_VS_ACT_IL4_AND_ANTI_IL12_48H_CD4_TCELL_DN	179	0.4875963	1.5188144	0.045908183	0.18632834	0.854	4092
GSE45739_NRAS_KO_VS_WT_UNSTIM_CD4_TCELL_UP	176	0.47568408	1.5177321	0.025641026	0.18715669	0.854	4573
GSE13547_CTRL_VS_ANTI_IGM_STIM_BCELL_2H_UP	168	0.5684265	1.5173186	0.044715445	0.18669531	0.854	3256
GSE3982_BASOPHIL_VS_TH2_DN	176	0.42655113	1.5108824	0.015936255	0.1977114	0.864	4267
GSE7460_CD8_TCELL_VS_TREG_ACT_DN	180	0.4276602	1.509204	0.045081966	0.1998671	0.869	2655
GSE24634_TREG_VS_TCONV_POST_DAY10_IL4_CONVERSION_UP	186	0.4818767	1.5034884	0.048484847	0.20769083	0.882	3315
GSE12845_IGD_POS_BLOOD_VS_DARKZONE_GC_TONSIL_BCELL_DN	174	0.38953394	1.5033928	0.01968504	0.2064171	0.882	3277
GSE13547_WT_VS_ZFX_KO_BCELL_DN	163	0.528856	1.5017288	0.034068137	0.20866154	0.884	3689
GSE37532_TREG_VS_TCONV_CD4_TCELL_FROM_LN_UP	180	0.5000738	1.5013813	0.048828125	0.20793039	0.885	4655
GSE22432_MULTIPOTENT_VS_COMMON_DC_PROGENITOR_UNTREATED_DN	177	0.44494435	1.5012527	0.035643563	0.20682704	0.885	4918
GSE12963_ENV_NEF_VS_ENV_NEF_AND_VPR_DEFICIENT_HIV1_INF_CD4_TCELL_DN	137	0.46060678	1.5006397	0.01417004	0.20701452	0.886	3033
GSE17974_2H_VS_72H_UNTREATED_IN_VITRO_CD4_TCELL_DN	169	0.45796275	1.4996994	0.036585364	0.2077742	0.887	3606
GSE12392_CD8A_POS_VS_NEG_SPLEEN_DC_DN	186	0.48802644	1.4985617	0.02811245	0.20742543	0.888	3740
GSE3982_BASOPHIL_VS_TH1_DN	181	0.44387075	1.4976739	0.025896415	0.20673086	0.888	4004
GSE16450_IMMATURE_VS_MATURE_NEURON_CELL_LINE_DN	180	0.5071201	1.4972848	0.048484847	0.20623013	0.889	4156
GSE41867_LCMV_ARMSTRONG_VS_CLONE13_DAY6_EFFECTOR_CD8_TCELL_DN	182	0.426203	1.4955812	0.046153847	0.20740704	0.894	5266
KAECH_NAIVE_VS_DAY8_EFF_CD8_TCELL_DN	187	0.54024214	1.4939747	0.033126295	0.20977426	0.896	3849
GSE25088_WT_VS_STAT6_KO_MACROPHAGE_IL4_STIM_DN	176	0.5213688	1.4929558	0.05	0.21085912	0.897	1859
GSE14908_RESTING_VS_HDM_STIM_CD4_TCELL_ATOPIC_PATIENT_UP	171	0.41765857	1.4917195	0.042596348	0.2123638	0.898	4332

GSE6259_FLT3L_INDUCED_33D1_POS_DC_VS_CD4_TCELL_DN	151	0.44271198	1.4912229	0.020242915	0.21209048	0.899	4177
GSE17974_0.5H_VS_72H_IL4_AND_ANTI_IL12_ACT_CD4_TCELL_DN	167	0.4385197	1.4889519	0.02173913	0.21477126	0.902	4391
GSE20727_CTRL_VS_ROS_INHIBITOR_TREATED_DC_UP	180	0.41056108	1.485857	0.029661017	0.21931219	0.905	4062
GSE40225_WT_VS_RIP_B7X_DIABETIC_MOUSE_PANCREATIC_CD8_TCELL_DN	174	0.41099095	1.4853885	0.02918288	0.21908581	0.905	4312
GSE9509_LPS_VS_LPS_AND_IL10_STIM_IL10_KO_MACROPHAGE_20MIN_DN	176	0.4982577	1.4839041	0.033663366	0.21997495	0.905	2993
GSE37532_WT_VS_PPARG_KO_LN_TCONV_DN	164	0.5147847	1.4838809	0.032323234	0.2187372	0.905	3416
GSE40068_BCL6_POS_VS_NEG_CXCR5_POS_TFH_UP	174	0.44974673	1.4833494	0.01002004	0.21730916	0.906	3736
GSE22432_CDC_VS_COMMON_DC_PROGENITOR_DN	177	0.4416654	1.483083	0.042596348	0.21666992	0.907	3276
GSE3982_MAC_VS_TH1_DN	183	0.4102568	1.4830233	0.017857144	0.2155361	0.907	2783
GSE2128_C57BL6_VS_NOD_THYMOCYTE_UP	177	0.40642616	1.4819562	0.029239766	0.21675958	0.909	3969
GSE3982_DC_VS_TH2_DN	182	0.4450947	1.4817274	0.018907564	0.21599461	0.909	3271
GSE17974_2.5H_VS_72H_IL4_AND_ANTI_IL12_ACT_CD4_TCELL_DN	166	0.4388875	1.4811851	0.02918288	0.21604872	0.91	4340
GSE30962_ACUTE_VS_CHRONIC_LCMV_SECONDARY_INF_CD8_TCELL_DN	184	0.46993825	1.4807215	0.031558186	0.2158128	0.911	2556
GSE29614_DAY3_VS_DAY7_TIV_FLU_VACCINE_PBMC_DN	162	0.46344477	1.4760576	0.036363635	0.22140844	0.916	3007
GSE40274_CTRL_VS_IRF4_TRANSDUCED_ACTIVATED_CD4_TCELL_DN	157	0.43749344	1.4756978	0.025052192	0.22094691	0.916	4528
GSE24671_BAKIMULC_VS_SENDAI_VIRUS_INFECTED_MOUSE_SPLENOCYTES_UP	175	0.45689175	1.473095	0.029940119	0.22345257	0.92	3871
GSE2770_UNTREATED_VS_IL4_TREATED_ACT_CD4_TCELL_2H_DN	178	0.43989345	1.4690725	0.044265594	0.22910345	0.928	3822
GSE3982_EOSINOPHIL_VS_TH1_DN	182	0.46934268	1.4687027	0.042682927	0.22885254	0.928	4227
GSE17301_IFNA2_VS_IFNA5_STIM_ACD3_ACD28_ACT_CD8_TCELL_DN	181	0.44320655	1.4671284	0.012096774	0.23124784	0.929	2778
GSE33425_CD8_ALPHAALPHA_VS_ALPHABETA_CD161_HIGH_TCELL_DN	182	0.48088357	1.4627864	0.04761905	0.23845069	0.932	3626
GSE16451_IMMATURE_VS_MATURE_NEURON_CELL_LINE_WEST_EQUINE_ENC_VIRUS_DN	183	0.44644076	1.4609526	0.038539555	0.23923728	0.934	4075
GSE19941_UNSTIM_VS_LPS_AND_IL10_STIM_IL10_KO_MACROPHAGE_DN	176	0.4303455	1.4599314	0.030674847	0.24055739	0.934	2912
GSE3982_NEUTROPHIL_VS_BCELL_DN	179	0.3951284	1.4547168	0.024590164	0.24934496	0.941	2912
GSE3720_UNSTIM_VS_PMA_STIM_VD2_GAMMADELTA_TCELL_DN	170	0.4327893	1.4544004	0.012048192	0.2489176	0.941	3226
GSE33425_CD161_HIGH_VS_INT_CD8_TCELL_DN	183	0.44298705	1.4535149	0.016260162	0.2485394	0.941	3626

Supp. Table S8 | Immunological signature gene sets enriched in hypermethylated *LRP2* stage II CC.

This table contains the most significant, positively enriched immune pathways in the hypermethylated *LRP2* (the 25% highest beta values for the cg13436799 CpG site; Q4) stage II CC subgroup ("Immunologic signature" gene sets, C7), obtained from the GSEA analysis. Abbreviations as follows: enrichment scores (ES), normalised enrichment scores (NES), nominal p values (NOM p-val) and false discovery rates (FDR q-val).

Supplementary Figures

Supplementary Figure S1:

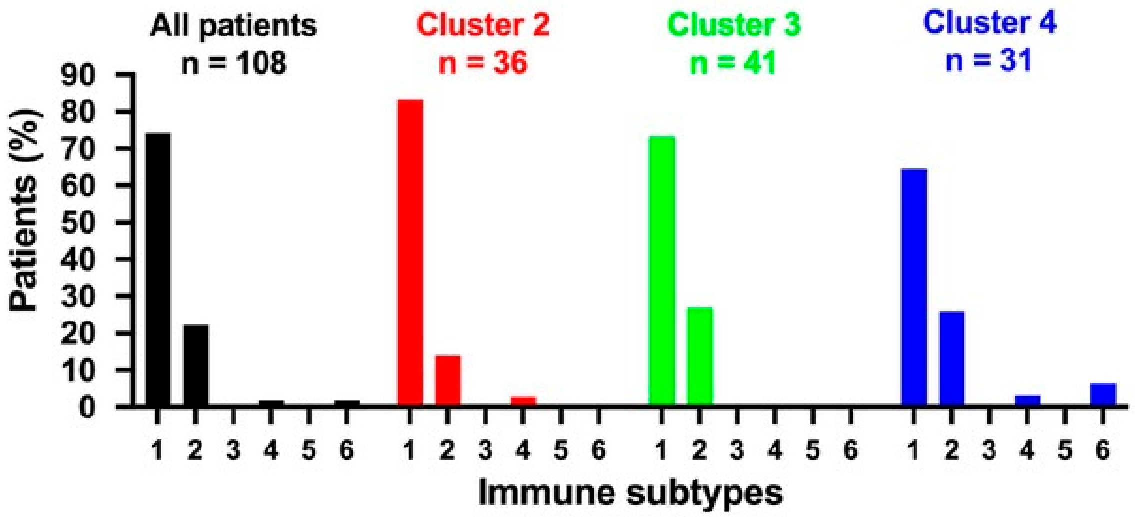

Supplementary Figure S1 | Immune landscapes by iAtlas analysis in the validation cohort of stage II CC from TCGA, according to CpG classifier group. Note the shift towards increased incidence of IFN- in clusters 3 and 4 compared to cluster 2 stage II CC patients and the presence of TGF- -dominant cases ('6') in cluster 4 patients. Percentage of CC tumours classified according to 6 different immune subtypes (numbered on X axis): 1= Wound Healing, 2= IFN-γ Dominant, 3= Inflammatory, 4= Lymphocyte Depleted, 5= Immunologically Quiet, 6= TGF-β Dominant (see text for details).

Supplementary Figure S2:

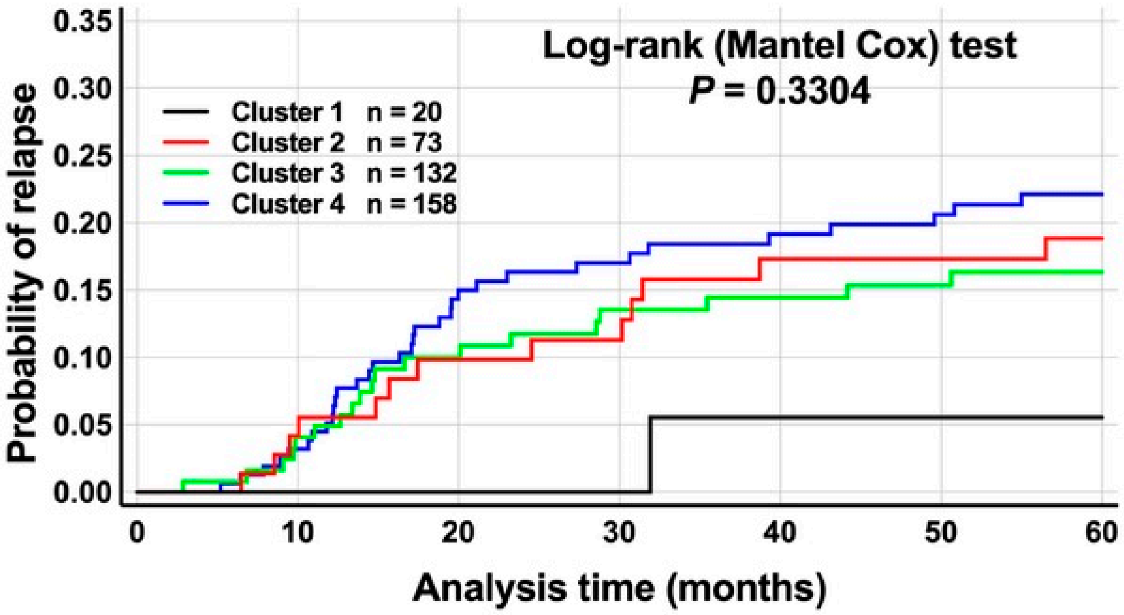

Supplementary Figure S2 | Estimation of the risk of relapse according to classification by DNA methylation cluster assignment in a population-based cohort of stage II colon cancer.
Cumulative probability of relapse for DNA methylation clusters 1 (black line), 2 (red line), 3 (green line) and 4 (blue line), respectively, during a 5-year follow-up period. The *p*-value of the Log-rank test is indicated.

Supplementary Figure S3:

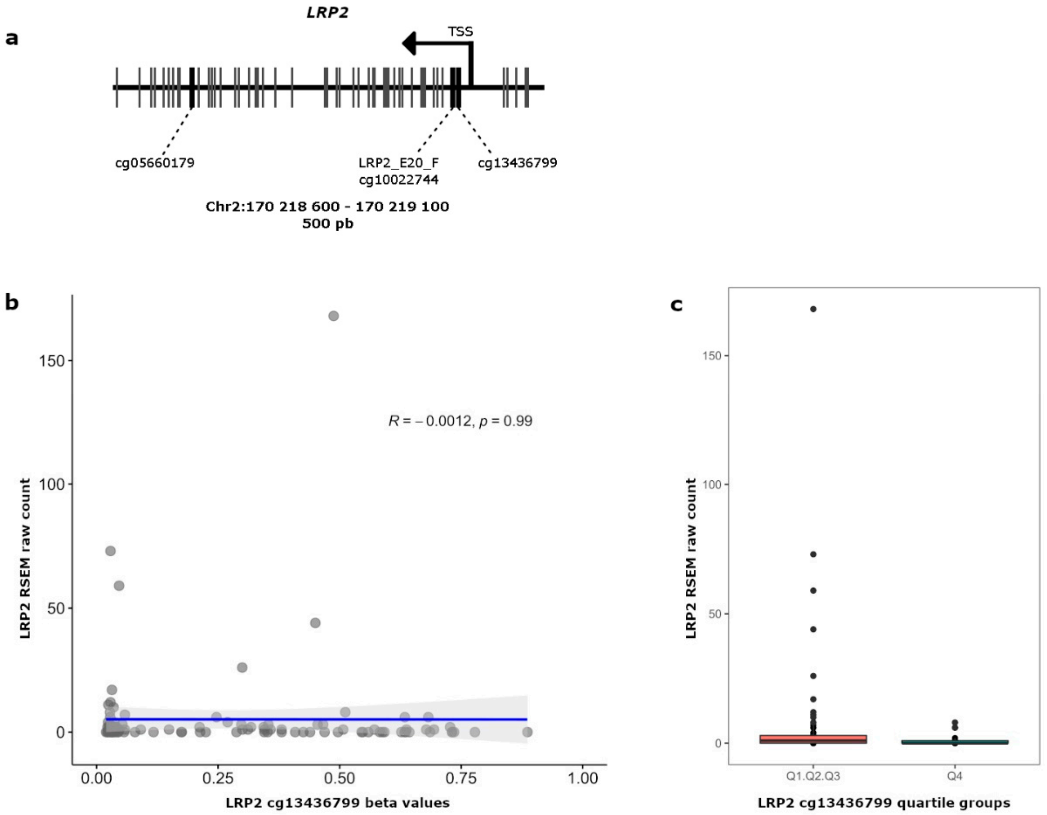

Supplementary Figure S3 | Correlation between DNA methylation and expression for *LRP2*
a, Map of the CpG site positions in the genomic region Chr2:170218600-170219100 (*LRP2* gene). CpG sites analysed in the study are indicated by thick black vertical lines. Other CpG sites are indicated by thin grey vertical lines**. b**, Linear regression plot between the RNAseq expression levels of *LRP2* (RSEM raw count) and DNA methylation levels (beta values) for the cg13436799 CpG site (data form the TCGA ; 108 stage II CC patients). The Pearson's correlation coefficient (*R*) and the null hypothesis probability (*p*) are indicated at the top of the plot. **c**, Box plot of *LRP2* RNAseq expression levels (RSEM raw count) for quartiles groups Q1/Q2/Q3 (the cases corresponding to 75% lower beta values for the cg13436799 CpG site) versus Q4 (the cases corresponding to the 25% higher beta values).

Supplementary Figure S4:

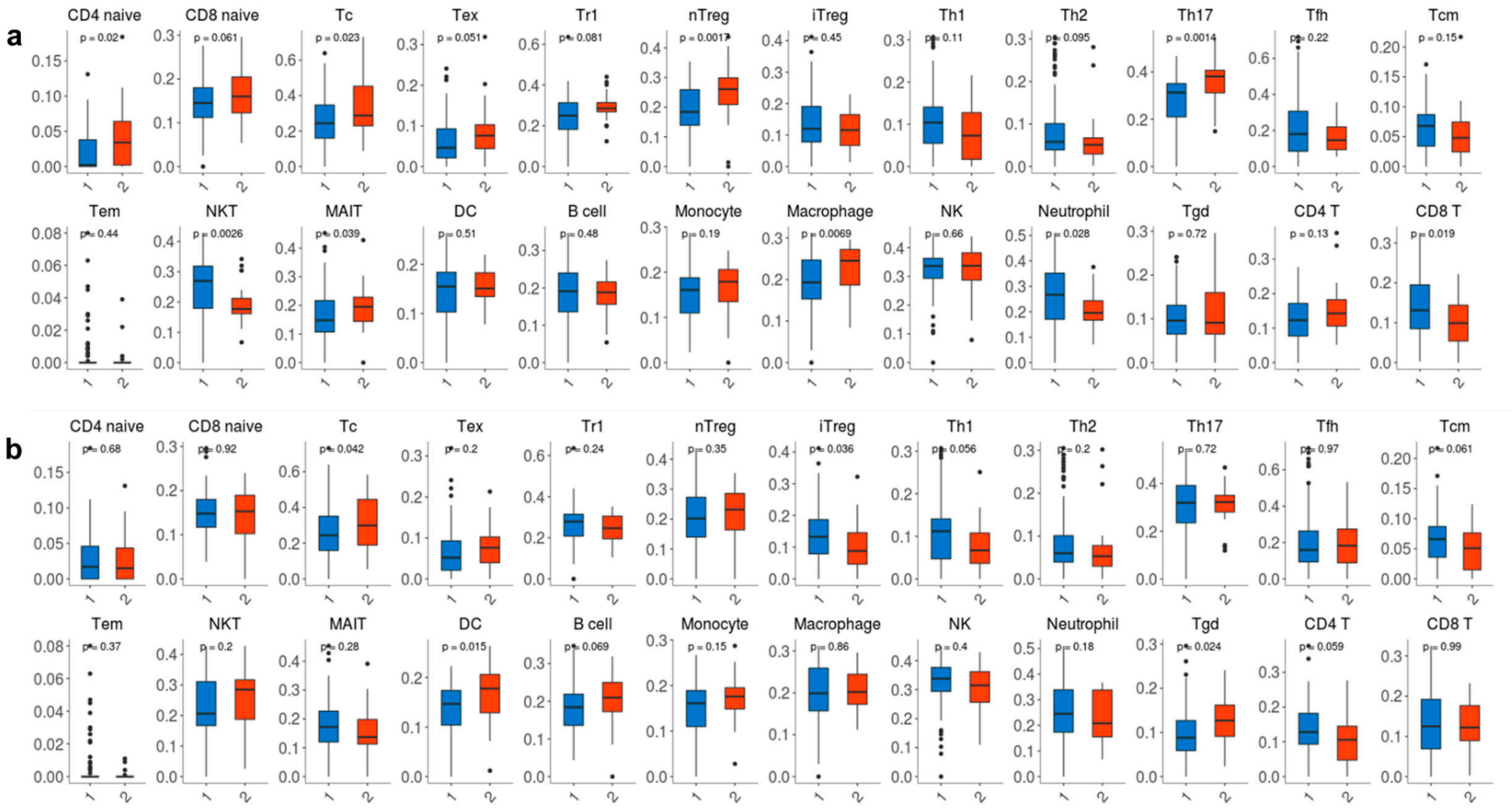

Supplementary Figure S4 | ImmuCellAI analysis of immune cell subsets in stage II CC patients (TCGA) from the validation cohort. In a, results for *CDH17* low (Q1-3; group 1, blue box plots) versus high methylation cases (Q4; group 2, red box plots). In b, results for *LRP2* low (Q1-3; group 1, blue box plots) versus high methylation cases (Q4; group 2, red box plots). See text for details. The names corresponding to abbreviations of cell population are the following: Tc: cytotoxic T cells; Tex: exhausted T cells; Tr1: Type 1 regulatory T cells; nTreg: natural regulatory T cells; iTreg: induced regulatory T cells; Th1: Type 1 T helper cells; Th2: Type 2 T helper cells; Th17: IL-17-producing effector T helper cells; Tfh: T follicular helper cells; Tcm: central memory T cells; Tem: effector memory T cells; NKT: natural killer T cells; MAIT: mucosal-associated invariant T cells; DC: Dendritic cells; NK: Natural killer cells; Tgd: Gamma delta T cells.

Supplementary Figure S5:

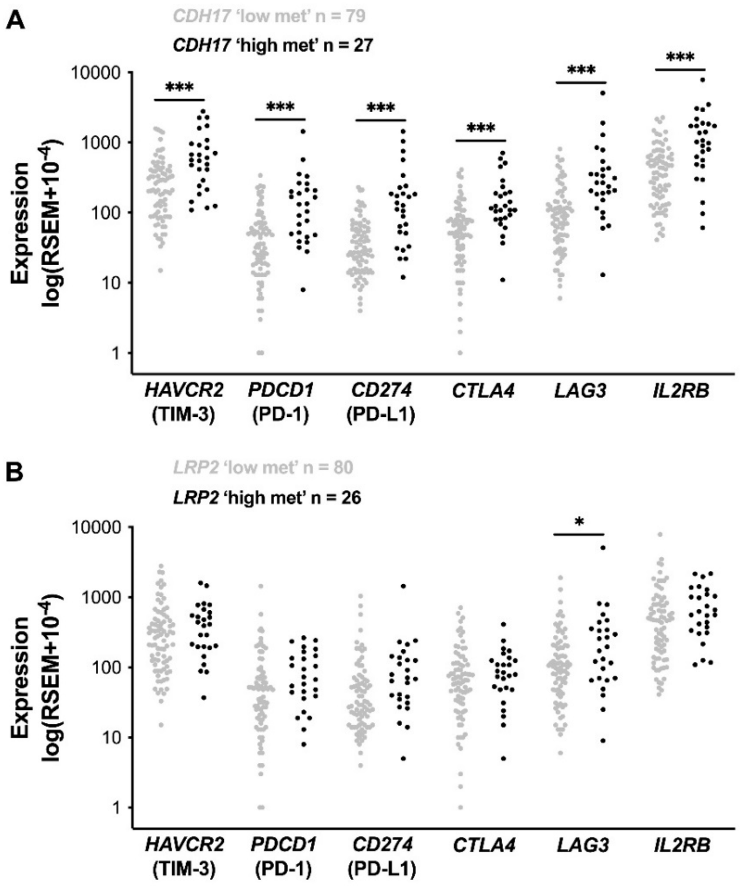

Supplementary Figure S5 |. Expression of genes related to immune escape according to *CDH17* and *LRP2* methylation status in stage II CC (TCGA). a, Scatter plot of RNAseq expression levels (log transformed RSEM raw count) between CDH17 'Low methylation' (quartiles groups Q1/Q2/Q3) versus CDH17 'High methylation' (Q4) subgroups. An unpaired Student's t-Test was performed and the resulting significance level of *p*-values between groups are indicated by asterisks (*: p-value ≤ 0.05, **: p-value ≤ 0.01, ***: p-value ≤ 0.001). **b,** Scatter plot of RNAseq expression levels between LRP2 'Low methylation' (quartiles groups Q1/Q2/Q3) versus LRP2 'High methylation' (Q4) subgroups.

Supplementary Figure S6:

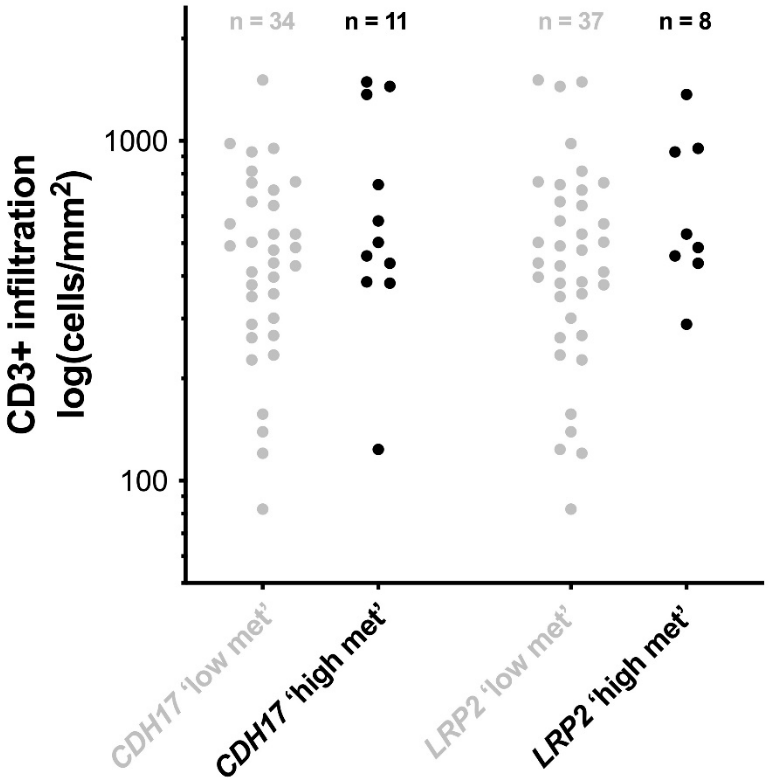

Supplementary Figure S6 | . Levels of CD3 positive lymphocytes in tumours according to *CDH17* and *LRP2* methylation status in stage II CC (Discovery cohort). Scatter plot of positive CD3 lymphocytes per surface (mm^2) between CDH17 'Low methylation' (quartiles groups Q1/Q2/Q3) versus CDH17 'High methylation' (Q4) subgroups or between LRP2 'Low methylation' (quartiles groups Q1/Q2/Q3) versus LRP2 'High methylation' (Q4) subgroups. An unpaired Mann-Whitney test was performed and the resulting significance level of *p*-values between groups are indicated by asterisks (*: p-value ≤ 0.05, **: p-value ≤ 0.01, ***: p-value ≤ 0.001).
